# A Branched Rutile/Anatase Phase Structure Electrode with Enhanced Electron-Hole Separation for High-Performance Photoelectrochemical DNA Biosensor

**DOI:** 10.3390/bios13070714

**Published:** 2023-07-07

**Authors:** Bingrong Wang, Bingdong Yan, Run Yuan, Bin Qiao, Guangyuan Zhao, Jinchun Tu, Xiaohong Wang, Hua Pei, Qiang Wu

**Affiliations:** 1 State Key Laboratory of Marine Resource Utilization in South China Sea, School of Materials Science and Engineering, Hainan University, Haikou 570228, China; brwang@hainanu.edu.cn (B.W.); yanbingdong@outlook.com (B.Y.); yr1550917260@163.com (R.Y.); wangxiaohong@hainanu.edu.cn (X.W.); 2 Department of Clinical Laboratory of the Second Affiliated Hospital, School of Tropical Medicine, Key Laboratory of Emergency and Trauma of Ministry of Education, Research Unit of Island Emergency Medicine, Chinese Academy of Medical Sciences (No. 2019RU013), Hainan Medical University, Haikou 571199, China; hy0211085@hainmc.edu.cn (B.Q.); zhaogy928@foxmail.com (G.Z.);

**Keywords:** PEC biosensor, branched titanium dioxide, anatase/rutile junction, CdTe-COOH QDs, DFT calculation

## Abstract

A photoelectrochemical (PEC) detection platform was built based on the branched rutile/anatase titanium dioxide (RA-TiO_2_) electrode. Theoretical calculations proved that the type-II band alignment of rutile and anatase could facilitate charge separation in the electrode. The self-generated electric field at the interface of two phases can enhance the electron transfer efficiency of the electrode. Carboxylated CdTe quantum dots (QDs) were applied as signal amplification factors. Without the target DNA presence, the CdTe QDs were riveted to the surface of the electrode by the hairpin probe DNA. The sensitization of CdTe QDs increased the photocurrent of the electrode significantly. When the target DNA was present, the structural changes of the hairpin probe DNA resulted in the failure of the sensitized structure. Benefiting from excellent electrode structure design and CdTe QDs sensitization strategy, the PEC assays could achieve highly sensitive and specific detection of target DNA in the range of 1 fM to 1 nM, with a detection limit of 0.23 fM. The electrode construction method proposed in this article can open a new avenue for the preparation of more efficient PEC sensing devices.

## 1. Introduction

Photoelectrochemical (PEC) biosensors have been universally regarded as a reliable tool for the analysis of various biomarkers, such as antigens [1,2,3], nucleic acids [4,5,6,7,8,9], enzymes [10,11], and some other chemicals [12,13,14]. PEC biosensors uniquely possess the advantages of low background signals, inherent miniaturization because of unique light excitation, and current detection pattern [15]. As a catalytic and transducer element, the photoactive electrode material plays an important role in the PEC biosensor. TiO_2_ is widely researched for electrode materials because of its chemical stability, photocatalytic activity, and biocompatibility [16,17,18,19]. However, electrodes prepared from TiO_2_ powder usually suffer from structural defects and charge recombination [20].

Branched TiO_2_ grown in situ on a conductive substrate has been proven to be an excellent photoanode for dye-sensitized solar cells [21,22] and PEC biosensing platforms [23,24,25]. For example, Yu et al. achieved ultra-sensitive photochemical DNA analysis using CdS/CdTe/TCPP co-sensitization structure and G-tetrad/heme catalysis based on branched TiO_2_ electrodes. However, the branched TiO_2_ electrodes reported in these studies consisted of single-phase rutile TiO_2_. The biggest problem with these electrodes is that poor charge separation in the single-phase rutile structure can seriously affect the signal collection ability of the electrode. Many scholars believe that this problem can be avoided by building a phase-structure electrode from different crystal forms of the same semiconductor. For example, the electrode composed of rutile and anatase TiO_2_ shows better photoelectrochemical properties than a single phase [26,27,28,29,30]. Therefore, constructing a phase-structure electrode may be an effective method for improving the performance of the PEC biosensor.

Although the phase-structure electrode can inhibit carrier recombination in the electrode, the photoelectric conversion efficiency of the electrode is not satisfactory because of the low light absorption in the visible range. This problem can be solved by using semiconductor QDs (like CdS QDs, CdSe QDs, and CdTe QDs) as a sensitizer [31]. Cadmium telluride (CdTe) is a known semiconductor nanomaterial with a bandgap of 2.0 eV, which is often used in PEC analysis systems as a signal amplification element. Combining CdTe QDs with the TiO_2_ can construct a sensitized structure to realize the amplification of electrode signals [32,33]. For example, Zhu et al. [31] achieved ultrasensitive PEC DNA detection based on a TiO_2_/CdS:Mn hybrid structure co-sensitized with two different sizes of CdTe QDs.

**Scheme 1:**
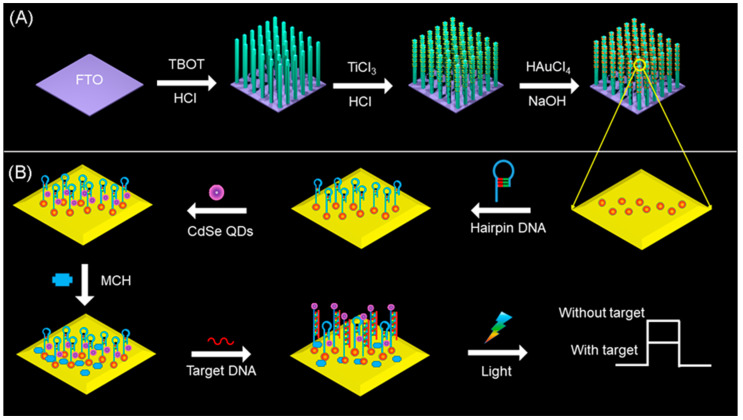
Schematic of (**A**) PEC electrode construction and (**B**) detection mechanism of target DNA.

Herein, a PEC DNA sensing platform was built based on an RA-TiO_2_ phase-structure electrode (Scheme 1). A phase structure was formed by assembling anatase TiO_2_ nanosheets onto rutile TiO_2_ nanorods. To detect target DNA sequences, the hairpin probe DNA was immobilized to the electrode surface by the Au-S bond. The sensitized CdTe QDs were connected to the head of hairpin DNA via the interaction of the -COOH and -NH_2_ groups. Given that the probe DNA is a hairpin structure, CdTe QDs were riveted on the electrode surface. The photocurrent response of the DNA sensor electrode was greatly enhanced by the sensitizing effect of CdTe QDs. Once the probe DNA hybridized with the target DNA, the probe DNA formed a rigid double-strand with the target DNA, forcing CdTe QDs away from the phase-structure electrode surface and leading to rapid decay of the electrode signals. As a result of the aid of the rutile/anatase phase-structure electrode, the electrochemical signals in the detection process could enter the electrode more efficiently and the target DNA could be sensitively detected.

## 2. Materials and Methods

### 2.1. Materials, Reagents, Apparatus

All reagents were used directly without purification. Fluorine-doped tin oxide (FTO; 1 cm × 2 cm) thin film substrate was obtained from Opivit New Energy Technology Co., Ltd. (Fushun, China). A glass cleaner was obtained from Shenzhen Run Ming Tong Technology Co., Ltd. (Shenzhen, China). Hydrochloric acid (HCl), tetrabutyl titanate (TBOT), titanium trichloride, ethanol, chloroauric acid (HAuCl_4_), cadmium chloride (CdCl_2_), sodium tellurite (Na_2_TeO_3_), 3-mercaptopropionic acid (MPA), ascorbic acid (AA), and sodium borohydride (NaBH_4_) were purchased from Sinopharm Chemical Reagent Co., Ltd. (Shanghai, China). 1-Ethyl-3-(3-(dimethylaminopropyl) carbodiimide hydrochloride (EDC), N-hydroxysuccinimide (NHS), Tris(2-carboxyethyl) phosphine hydrochloride (TCEP), and 6-mercapto-1-hexanol (MCH) were supplied by Sigma-Aldrich. Tris-hydroxymethylamino methane-hydrochloride (Tris) buffer solution was supplied by Shanghai Roche Pharma Co., Ltd. (Shanghai, China). The sequences of oligonucleotides fabricated by Sangon Biotech Co., Ltd. (Shanghai, China) are listed in Table 1.

The morphology of the samples was characterized by field-emission scanning electron microscopy (FESEM, Hitachi SU8010). Transmission electron microscopy (TEM) images were obtained using TEM (JEOL JEM 2100). The XRD patterns of the as-prepared films were recorded on a Philip X’pert X-ray diffractometer (PANalytical, Almelo, The Netherlands; CuKa irradiation, λ = 0.15418 nm) from 5° to 80° at a scanning speed of 10°/min. UV–vis absorption spectra were obtained on a UV–vis absorption spectrophotometer (UV–vis, Thermo Fisher Evolution 220). PL and TRPL measurements were made on an FL3-P-TCSPC time-resolved fluorescence spectrometer (Horiba Jobin Yvon, Longjumeau, France). The test was based on TiO_2_ NRs, with 425 nm as the emission wavelength. The highest excitation wavelength (330 nm) was selected as the characteristic excitation wavelength, and the emission spectrum in the wavelength range of 350–800 nm was recorded. PEC tests were performed with a homemade PEC system. A xenon lamp (Wavelength range: 200–1000 nm, Beijing Ceaulight Technology Co., Ltd., Beijing, China) served as the irradiation source. Photocurrent was recorded on an electrochemical workstation (Chenhua Technology Co., Ltd., Shanghai, China) by a three-electrode electrochemical system with Ag/AgCl reference electrodes and 0.5 cm × 0.5 cm Pt foil as counter electrodes under an off-on-off (10 s-10 s-10 s) switching light at 0.0 V in the electrolyte of 0.1 M PBS (containing 0.1 M AA, pH 7.4). Electrochemical impedance spectroscopy (EIS) was performed on a Bio-Logic electrochemical workstation (France) in a 0.1 M KCl solution containing 5.0 mM K_3_[Fe(CN)_6_]/K_4_[Fe(CN)_6_] (1:1) at an open circuit potential over a frequency range of 10 mHz–100 kHz with 5 mV AC perturbation.

### 2.2. Synthesis of the R-TiO_2_ NR Electrode

Rutile TiO_2_ NRs were grown in-situ on FTO glass substrate according to the literature with slight modification [3]. About 400 μL of tetrabutyl titanate was mixed with diluted HCl aqueous solution, which contained 10 mL of H_2_O and the same volume of concentrated HCl (37%). The mixture was stirred until clear and poured into a Teflon liner. The FTO substrates were cleaned ultrasonically with cleaning agent and settled vertically in a Teflon liner. The liner was placed in a stainless-steel autoclave and heated in an oven at 150 °C for 4 h. R-TiO_2_ NR electrodes were obtained by annealing the substrate at 450 °C in air atmosphere.

### 2.3. Fabrication of RA-TiO_2_ Phase-Structure Electrodes

RA-TiO_2_ phase-structure electrodes were fabricated by a sequential hydrothermal process [28]. In a typical experiment, 0.25 mL of TiCl_3_ was added dropwise to a mixed solution prepared from 0.25 mL of concentrated HCl and 20 mL of deionized water. The R-TiO_2_ NR electrodes were placed into the Teflon liner. After the mixed solution was poured in, the Teflon liner was placed in an autoclave and heated at 80 °C for 1 h. RA-TiO_2_ phase-structure electrodes were obtained after the substrates were removed and annealed at 300 °C for 3 h in a muffle furnace.

### 2.4. Synthesis of Carboxylated CdTe QDs

Typically, 120 mL of DI water was poured into a three-necked flask. After the solution was deoxygenated with N_2_, 0.6 mmol CdCl_2_ and 1.02 mmol MPA were dissolved into the aqueous solution. Subsequently, 0.1 M NaOH was added to the mixture until the pH was 11.8. About 120 mg of NaBH_4_ and 20 mg of Na_2_TeO_3_ were successively dissolved into the deoxygenated alkaline mixture. The mixture solution was refluxed under N_2_ protection for 6 h at 100 °C to prepare a carboxylated CdTe QD solution.

### 2.5. PEC Assay Procedure

The FTO/TiO_2_/Au electrodes were prepared by dipping RA-TiO_2_ phase-structure electrodes into 0.01% HAuCl_4_ (pH 4.5) for 30 min. After pretreatment with TCEP (100 mM) for 1 h, probe DNA (20 μL, 1 μM) was incubated with the FTO/TiO_2_/Au electrodes at 4 °C in the fridge overnight. The prepared electrode was rinsed in TE (Tris-EDTA buffer) buffer to remove unbound probe DNA. CdTe QDs were activated by 200 μL of 20 mg/mL EDC and 10 mg/mL NHS solution for 0.5 h. The electrodes were incubated with 20 μL of carboxylated CdTe QD solution for 1 h at 37 °C. To block non-specific binding sites, 20 μL of 1 mM MCH was incubated with the prepared electrode for 1 h. The electrode was incubated with 20 μL of different concentrations of the target DNA at 37 °C for 1 h for further PEC tests.

### 2.6. Theoretical Calculations

First-principles calculations were performed using the Vienna ab initio simulation package (VASP) within the density functional theory (DFT) framework. The generalized gradient approximation was performed on the models with the Perdew–Burke–Ernzerhof exchange-correlation potential and the projector-augmented wave method to calculate the exchange-correlation energy functional. The structural and electronic properties of bulk rutile and anatase were calculated on a 2 × 2 × 1 k-point mesh with a cutoff energy of 500 eV. As for the rutile (110)/anatase (101) heterojunction, the K points of 2 × 2 × 1 were set for geometry optimization and the cutoff energy was set to 500 eV for a plane wave basis. The thickness of vacuum layers was set to 20 Å along the surfaces of all the models to avoid influences of neighboring slabs. Maximum residual forces of the models were relaxed until there was less than 0.01 eV/Å on each atom.

## 3. Results

### 3.1. Composition and Morphology Characterization of the Electrodes

**Figure 1:**
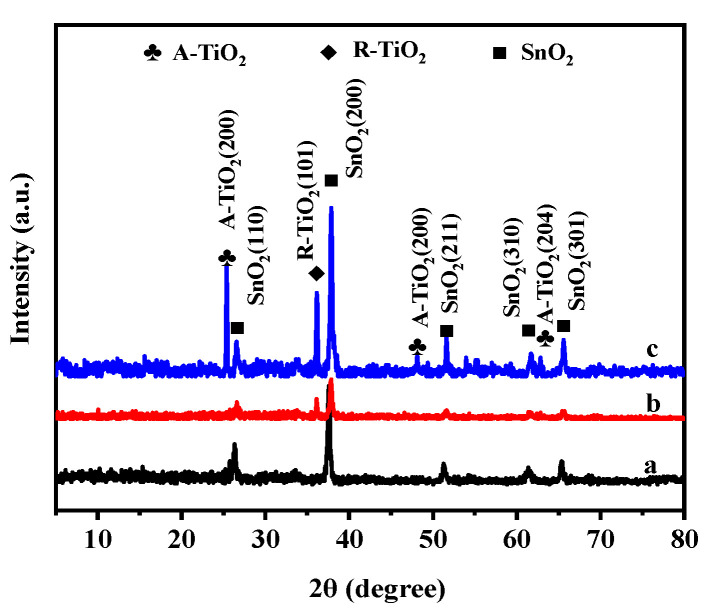
X-ray diffraction patterns of the (a) FTO; (b) R-TiO_2_ NR and (c) branched RA-TiO_2_ phase-structure electrodes.

The composition of the electrodes was investigated by X-ray diffraction (XRD). As shown in Figure 1, the FTO glass showed peaks at 26.3°, 37.6°, 51.3°, 52.9°, and 59.9°, which were consistent with SnO_2_ (JCPDS No.41-1445). The hydrothermally grown electrode showed new diffraction peaks located at 36.2°, which were assigned to the rutile TiO_2_ (101) diffraction peaks in accordance with JCPDS No.21-1276, indicating that the crystalline form of the NRs was rutile TiO_2_. The same diffraction patterns of rutile TiO_2_ and SnO_2_ were found in the branched RA-TiO_2_ phase-structure electrode. In addition, new characteristic peaks at 25.5° were found in the XRD patterns, indicating that the crystalline form of nanosheets was anatase TiO_2_, which was in accordance with JCPDS No. 21-1272. In conclusion, the XRD results show that the electrodes are composed of rutile and anatase.

**Figure 2:**
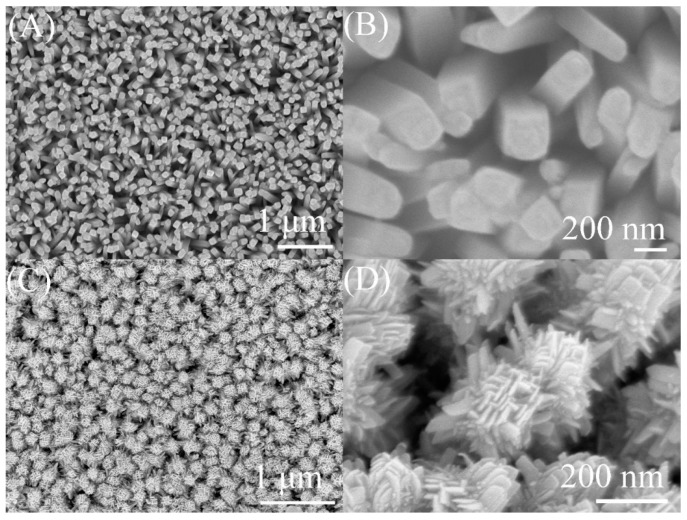
(**A**) FESEM images and (**B**) high-resolution FESEM image of the R-TiO_2_ NR electrode; (**C**) FESEM images and (**D**) high-resolution FESEM image of the branched RA-TiO_2_ phase-structure electrode.

The surface morphology of the sequential hydrothermal synthesized electrodes is displayed in Figure 2. Figure 2A shows the top view of the hydrothermally grown electrode; the substrate was covered with uniform rod-like nanostructures. High-resolution FESEM images of the TiO_2_ NRs are presented in Figure 2B. These TiO_2_ NRs were columnar cuboids topped by a square with a width of ~100 nm and were tightly grown on the substrate. Figure 2C presents the top view of the sequential hydrothermal synthesized electrode. Numerous small nanosheets had grown on the top of the NRs to form branched nanostructures after sequential hydrothermal synthesis. As we can see from the high-resolution FESEM image of the branched RA-TiO_2_, the TiO_2_ NRs and nanosheets formed the trunks and leaves of the tree-like nanostructures (Figure 2D).

**Figure 3:**
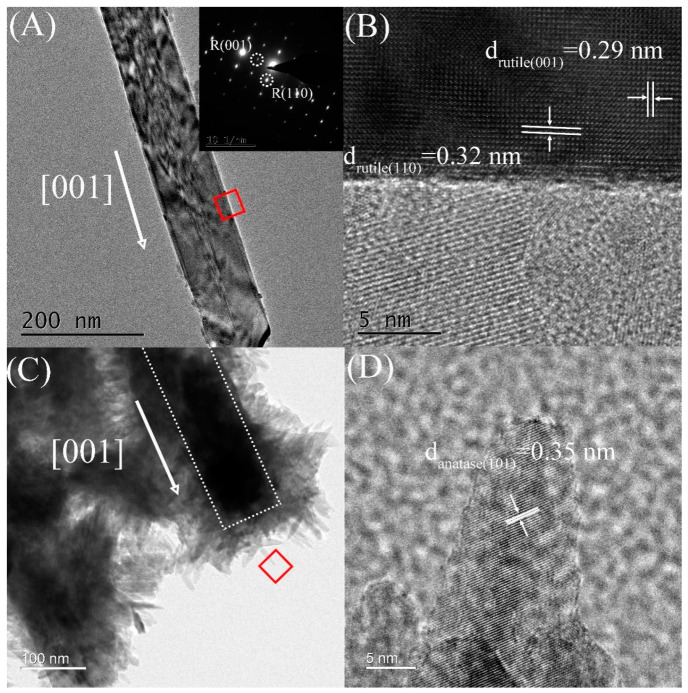
(**A**) TEM image and (**B**) HRTEM image of the R-TiO_2_ NR electrode; (**C**) TEM image and (**D**) HRTEM image of the branched RA-TiO_2_ phase-structure electrode.

The crystallographic information of the rutile TiO_2_ NRs and the branched RA-TiO_2_ phase-structure are shown in Figure 3. The TEM image of rutile TiO_2_ NR in Figure 3A shows a typical rod-like structure. The inset of Figure 3A shows the selected-area electron diffraction patterns (SAED) of rutile TiO_2_ NRs. The bright and periodic spots in SAED indicate the single-crystalline feature of the rutile TiO_2_ nanorods. The HRTEM images of rutile TiO_2_ NR in Figure 3B showed clear crystalline lattice fringes. The lattice spacings of the NRs were 0.32 and 0.29 nm, corresponding to {110} and {001} of rutile, respectively [34]. The results of electron diffraction patterns and lattice fringes indicate that the growth direction of rutile nanorods is [001]. The TEM image of the branched RA-TiO_2_ nanostructure is presented in Figure 3C, showing a large number of nanosheets grown around the rutile TiO_2_ NRs. The HRTEM images in Figure 3D of the branched RA-TiO_2_ show a lattice spacing of 0.35 nm, corresponding to the {101} of anatase TiO_2_. In brief, the (101) facet of anatase nanosheets was in contact with the (110) facet of rutile NRs. Given that anatase crystals have higher photocatalytic activity than rutile crystals, branched RA-TiO_2_ nanostructures with anatase as the external layer can multiply the photocatalytic active sites [35].

### 3.2. PEC Analysis of the Electrode

**Figure 4:**
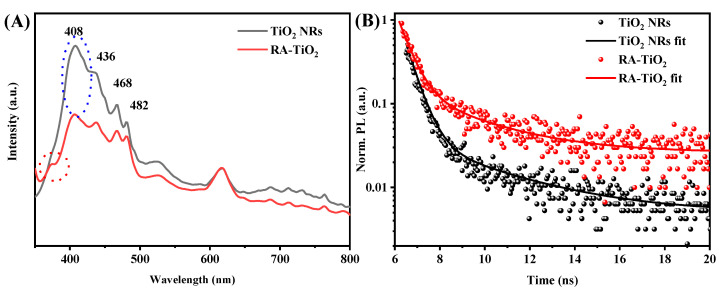
(**A**) Photoluminescence spectroscopy. (**B**) Time-resolved photoluminescence spectroscopy of TiO_2_ NRs electrode and branched RA-TiO_2_ phase-structure electrode.

To prove that a branched RA-TiO_2_ phase structure can inhibit the recombination of photogenerated charges, a series of photoluminescence (PL) tests was carried out to reveal the efficiency of electron–hole recombination. Four obvious peaks at 408, 436, 468, and 482 nm are shown in Figure 4A, which were equivalent to energies of 3.04, 2.84, 2.64, and 2.57 eV, respectively. The characteristic peak at 408 nm (blue oval), the energy of which was nearly equal to 3.0 eV, was attributed to the interband photoluminescence of rutile TiO_2_ [27]. Compared with the R-TiO_2_ NR electrode, the emission intensity of the branched RA-TiO_2_ phase-structure electrodes in the range of 400–500 nm decreased significantly, indicating that the branched RA-TiO_2_ phase structure could inhibit the recombination of photogenerated charges. Notably, an obvious characteristic peak (red oval) of anatase TiO_2_ at 387 nm, the energy of which was nearly equal to 3.2 eV, indicated that the nanosheets were anatase phase [27].

Time-resolved photoluminescence spectroscopy (TRPL) was performed to investigate the lifetime of the photogenerated electrons in the R-TiO_2_ NR electrode and the branched RA-TiO_2_ phase-structure electrode. The corresponding electron–hole pair lifetimes were calculated by fitting the data with a biexponential decay function. As shown in Figure 4B, the average lifetime (τ_ave_) values for the TiO_2_ NRs and RA-TiO_2_ samples were 1.51 ns and 8.02 ns, respectively. The fitted parameters are listed in Table 2. The results of the TRPL indicated that the branched RA-TiO_2_ phase structure could prolong the photogenerated carrier’s lifetime, which would greatly strengthen the catalytic ability of the electrode.

### 3.3. Theoretical Calculation

**Figure 5:**
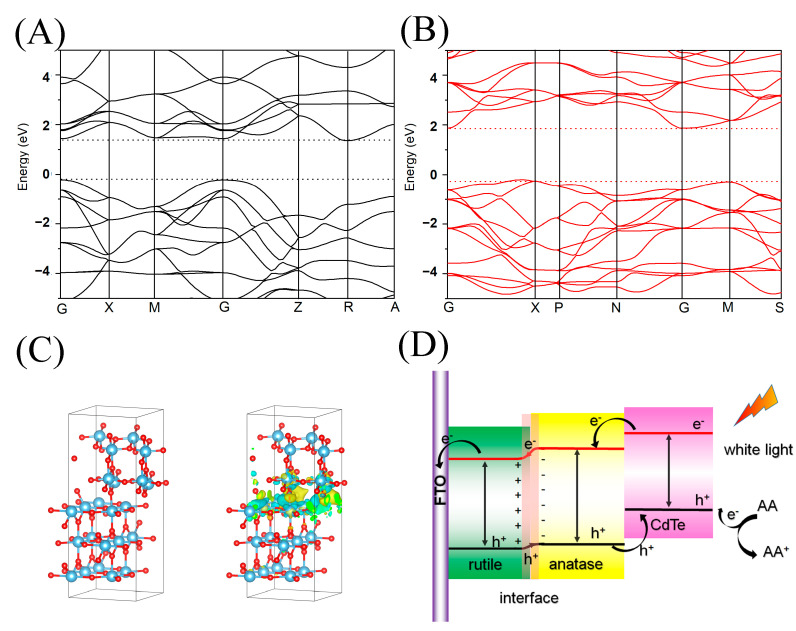
Band structure of (**A**) the rutile and (**B**) anatase phases. (**C**) Calculation model (left) and differential charge density (right) between the interface of rutile (110) and anatase (101) (Ti, blue balls; O, red balls). (**D**) Electron transfer mechanism of the DNA biosensor.

DFT calculations were performed to study the band structure of the rutile and anatase phases and charge distribution within the branched RA-TiO_2_ phase-structure electrode. In Figure 5A,B, the conduction band bottom of the rutile phase was lower than that of the anatase phase by ~0.5 eV, whereas the valence band top was similar. The band arrangement of the electrodes showed the characteristic of II-type heterojunction. To demonstrate the role of the branched RA-TiO_2_ phase-structure electrode in signal collection, the differential charge density at the interface between rutile (110) and anatase (101) was simulated. As shown in Figure 5C, the calculation models were built by cutting rutile TiO_2_ crystal along with rutile TiO_2_ (110) and anatase TiO_2_ (101). The results of differential charge density of the model demonstrated that electrons gathered at the anatase (101) surface, whereas holes gathered at the rutile (110) surface and brought them together. The self-built electric field was established at the interface to provide a driving force for the collection of electrode signals. Therefore, as shown in the electron transfer diagram in Figure 5D, when there was no target in the detection system, the hairpin probe DNA riveted CdTe QDs on the surface of the electrode. The TEM images presented in Figure S1 reveal that the CdTe QDs were regular spherical structures with diameters of about 2–5 nm and the SEAD diffraction pattern indicates that the CdTe QDs were polycrystalline powders. The photoexcited electrons from the CdTe QD conduction band entered the conduction band of anatase TiO_2_ and were then received by the FTO electrode through the rutile NRs. The formation of the rutile/anatase/CdTe-sensitized structure significantly improved the photoelectric response of the electrode. However, the hairpin probe DNA hybridized with the target DNA to form a hard-to-bend double-stranded structure, forcing CdTe QDs off the electrode surface and resulting in a significant decrease in electrode photocurrent. With the aid of the branched RA-TiO_2_ phase-structure electrode, the electrochemical signals in the detection process could enter the electrode more smoothly and efficiently.

### 3.4. Characterization of the PEC Biosensor

**Figure 6:**
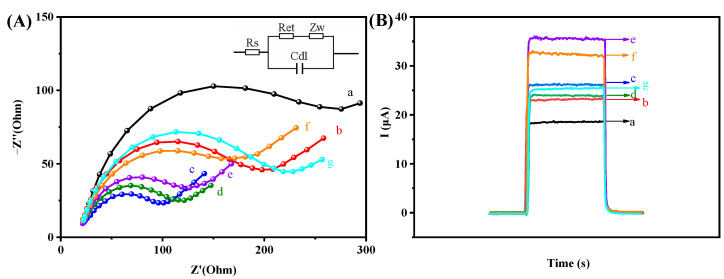
(**A**) Electrochemical impedance spectroscopy and (**B**) photocurrent of (a) the R-TiO_2_ NR electrode and (b) branched RA-TiO_2_ phase-structure electrode (c) after Au deposition and (d) after incubation with hairpin probe DNA, (e) CdTe QDs, (f) MCH, and (g) 20 μL of target DNA (1 nM).

The stepwise assembly process in the PEC biosensor was studied by electrochemical impedance spectroscopy (EIS) and photoresponse current–time tests. The EIS spectrum of the electrode shown in Figure 6A was composed of a semicircular component and a linear component, which were ascribed to the electron transfer-limited process and diffusion-limited process, respectively [36]. A small semicircle diameter represents low resistance to electron transfer. The R-TiO_2_ NR electrode showed a large impedance semicircle (curve a, R_et_ = 329 Ω). When anatase nanosheets were grown on the rutile NR array, the electrode impedance was further reduced (curve b, R_et_ = 246 Ω). The internal electric field accelerated electron transfer, which resulted in the decrease in electrode impedance. The impedance of the electrode was greatly reduced when the AuNPs with good conductivity were deposited (curve c, R_et_ = 115 Ω). R_et_ increased after incubation with probe DNA, CdTe QDs, and mercaptohexanol (MCH) (curve d, R_et_ = 139 Ω; curve e, R_et_ = 171 Ω, curve f, R_et_ = 221 Ω) because of the electrostatic repulsion among the [Fe(CN)_6_]^−3/−4^ group to the phosphate group on DNA, the carboxylic acid group on the CdTe QD surface, and the hydroxyl group on mercaptohexanol. The introduction of target DNA led to the further increase in R_et_ (curve g, R_et_ = 243 Ω), which was ascribed to the formation of double strands, resulting in a decrease in the conductivity of the nucleic acid strand and indicating the successful hybridization of the probe and the target DNA.

The photoelectric current responses at different stages of the electrode are recorded in Figure 6B. The photocurrent of the R-TiO_2_ NR electrode was ~18 μA (curve a). When anatase nanosheets were grown on the R-TiO_2_ NR electrode, the photocurrent value of the electrode further increased (curve b, I = 24 μA). After Au deposition, the photocurrent increased slightly (curve c, I = 26 μA) due to the surface plasmon resonance effect of Au NPs [37]. The photocurrent decreased slightly (curve d, I = 23 μA) after hairpin probe DNA immobilization, which was ascribed to the poor conductivity of the nucleotide chain. The photocurrent increased sharply after incubation with CdTe QDs (curve e, I = 36 μA) due to the sensitization effect of the rutile/anatase/CdTe QD structure. A slight decrease was noted in the photoelectric response current of the electrode when the end-capping agent MCH was loaded onto the electrode surface (curve f, I = 33 μA), which was attributed to the poor conductivity of MCH. The photocurrent of the electrode decreased after incubation with the target DNA (curve g, I = 25 μA), indicating successful hybridization of the probe with the target DNA. Combining the results of EIS and photocurrent response, the successful fabrication of the PEC platform was confirmed.

### 3.5. Analysis Performance of PEC Biosensor

**Figure 7:**
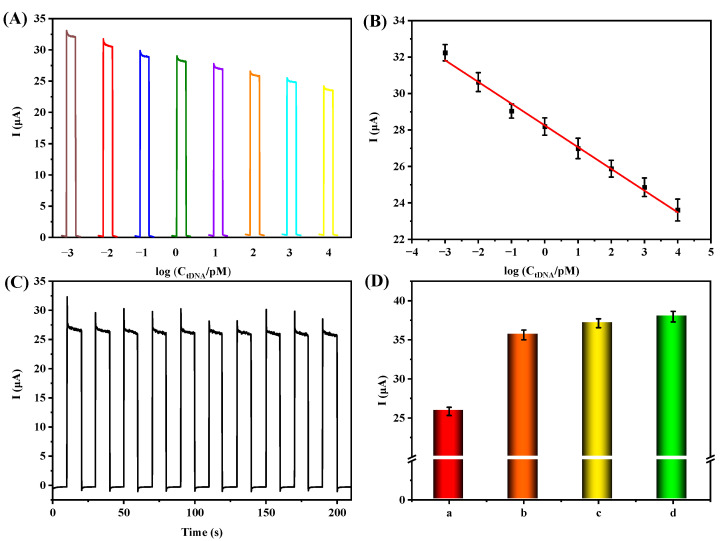
(**A**) Photocurrent signals of the sensing platform with different concentrations of target DNA, (**B**) relationship of photocurrent versus logarithm of target DNA concentration, (**C**) stability test of the electrode with 10 μM target DNA, and (**D**) selectivity test of the electrodes.

To explore the impact of the concentration of CdTe QDs and incubation time on our detection performance, we performed a parameter adjustment experiment. The results are presented in Figure S2. The optimal concentration of CdTe QDs was 5 mg/mL and the incubation time was 60 min. In Figure 7A, as the sensitization effect vanished due to target DNA hybridization, the photocurrent decreased with the increase in the target DNA concentration. The relationship of photocurrent versus logarithm of target DNA concentration in the range of 1 fM–10 nM is presented in Figure 7B. The linear regression equation was I = 28.25–1.19 log *c* (*R*^2^ = 0.9951). The limit of detection (LOD) for the target DNA concentration was calculated as 23 fM (*S/N* = 3). To reflect the sensitivity of the PEC sensing platform, we compared the biosensing performance of the PEC platform with other DNA detection methods. As shown in Table 3, the platform we proposed provides a low LOD in a wide detection range.

To verify the stability of the electrode, the photocurrent was tested with 10 μM target DNA under 10 on/off illumination cycles. No significant change was observed during the test cycles (Figure 7C, calculated relative standard deviation RSD = 5.64%), which indicated the good reproducibility of this electrode. Additionally, the selectivity was investigated by testing the photocurrent of the electrode with 10 μM target DNA, 10 μM single-base mismatch DNA, 10 μM noncomplementary DNA, and a blank sample (Figure 7D). The photocurrent of the electrode with 10 μM target DNA (column a, I = 25.84 μA) decreased significantly more than the photocurrent of the blank sample (column d, I = 37.69 μA), whereas the single-base mismatch (column b, I = 35.62 μA) and noncomplementary DNA (column c, I = 37.12 μA) were very close to the blank sample. These results showed that the proposed DNA sensing electrode could offer satisfactory specificity. To evaluate the applicability of the electrode, recovery was analyzed by adding different amounts of target DNA (1.0, 10, and 100 pM) to human serum samples (Table S1). The recovery of target DNA in the serum samples was in the range of 95.18–100.64% and the RSD was less than 3.9%, indicating potential applications of the biosensing electrode in complex samples.

## 4. Conclusions

In summary, a PEC sensing platform was fabricated based on branched rutile/anatase TiO_2_ phase-structure electrodes. As expected, compared with the single-phase TiO_2_ NR electrode, the proposed sensor possessed a higher photoelectric response, a lower carrier recombination rate, and a longer carrier lifetime. The proposed PEC sensing electrode could offer an LOD of 0.23 fM in a wide linear range between 1 fM and 1 nM. These high PEC performances are related to the improved separation ability of the photogenerated charges due to the branched RA-TiO_2_ phase structure, a self-built electric field caused by the interface design between the two phases, and a reasonable signal amplification strategy based on CdTe QD sensitization.

## Figures and Tables

**Table 1:** Specific oligonucleotide sequences.

Oligonucleotide	Sequences (From 5′ to 3′)
Hairpin DNA	5′-H_2_N-(CH_2_)_6_-CTC GCT TGG AAT AGC TGT GAT CAT TGT TAT TAG CGA GTT T-(CH_2_)_6_-SH-3′
Target DNA	5′-CTC GCT AAT AAC AAT GAT CAC AGC TAT TCC A-3′
Single-base mismatch	5′-CTC GCT AAT AAC AAT TAT CAC AGC TAT TCC A-3′
Noncomplementary	5′-TAT ATC TGA TCT GTC CCA ATT GTA CGA GTA T-3′

**Table 2:** Decay parameters and average lifetime according to a biexponential fitting model of the PL decay curves obtained from the samples.

Samples	τ_1_	τ_2_	A_1_	A_2_	τ_ave_
R-TiO_2_	0.48	3.63	428.13	27.36	1.51
RA-TiO_2_	0.63	13.76	680.45	40.17	8.02

**Table 3:** Comparison of different sensors for the detection of target DNA.

Biosensors	Dynamic Range	Detection Limit	References
FL ^1^	0.01 pM–10 nM	3 pM	[38]
DPV ^2^	10 fM–0.1 μM	0.31 fM	[39]
ECL ^3^	50 fg/mL–500 ng/mL	32 fg/mL	[40]
EC ^4^	0.01 pM–10 nM	3.5 fM	[41]
PEC	10 fM–0.1 μM	3 fM	[42]
PEC	1 fM–1 nM	0.23 fM	This work

^1^ fluorescence; ^2^ differential pulse voltammetry; ^3^ electroluminescence; ^4^ electrochemistry.

## Data Availability

Not applicable.

## Supplementary Materials

The following supporting information can be downloaded at: https://www.mdpi.com/article/10.3390/bios13070714/s1, Figure S1: TEM image and SEAD of CdTe QDs; Figure S2: Effect of concentration of CdTe QDs and incubation time with target DNA on the photocurrent response; Table S1: Analytical results of target DNA in human serum samples using the proposed method.
