# Supplementary material for: A Branched Rutile/Anatase Phase Structure Electrode with Enhanced Electron-Hole Separation for High-Performance Photoelectrochemical DNA Biosensor
Source: Biosensors (Basel). 2023 Jul 7;13(7):714. doi: 10.3390/bios13070714 (PMC10377446; doi:10.3390/bios13070714)
Supplement: Supplementary file 1 [file biosensors-13-00714-s001.zip › biosensors-2477352-supplementary.pdf]

A Branched Rutile/Anatase Phase Structure Electrode with Enhanced Electron-Hole Separation for High-Performance Photoelectrochemical DNA Biosensor

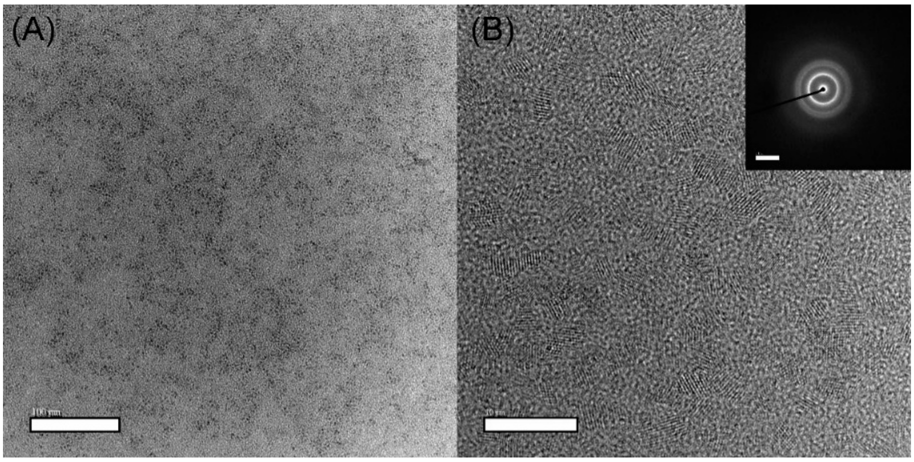

Figure S1. (A, B, inset) TEM image and SEAD of CdTe QDs

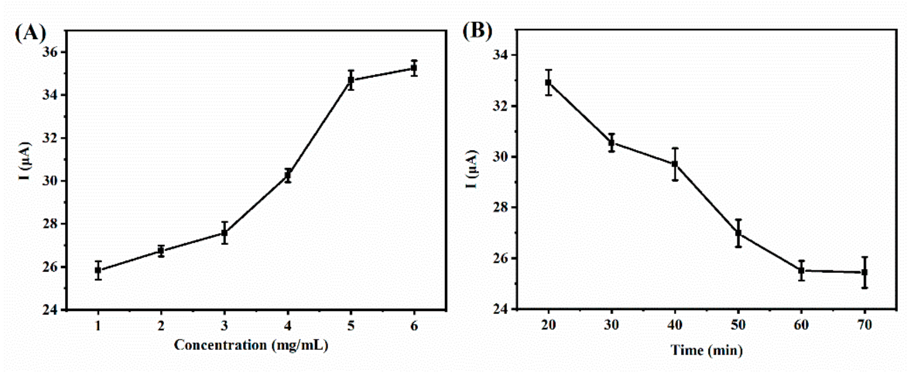

Figure S2. (A) Effect of concentration of CdTe QDs and (B) incubation time with target DNA on the photocurrent response

Table S1. Analytical results of target DNA in human serum samples using the proposed method

Human serum	Cadded (pM)	Cfound (pM)	RSD% (n=3)	Recovery (%)
Sample 1	1	0.9418	2.6	95.18
Sample 2	10	9.821	3.9	96.21
Sample 3	100	100.42	3.5	100.64
